# Effects of Yangsaeng (Health Management) Therapy for Korean Older Adults in Nursing Home

**DOI:** 10.3390/ijerph17207507

**Published:** 2020-10-15

**Authors:** Sohyune R. Sok, Seyoon Kim, Da Un Jeong, Youngmi Cho

**Affiliations:** 1 College of Nursing Science, Kyung Hee University, Seoul 02447, Korea; 2 Department of Nursing, Graduate School, Kyung Hee University, Seoul 02447, Korea; ssamyun85@naver.com (S.K.); mynameisjdw@naver.com (D.U.J.); 3 Department of Nursing, Choonhae College of Health Sciences, Ulsan 44610, Korea; choyoung23@yahoo.com

**Keywords:** aged, Yangsaeng, nursing home

## Abstract

The number of older adults admitted to nursing homes is steadily increasing, and the health management for them is a very important issue in Korean society. This study aimed to examine the effects of Yangsaeng (health management) therapy on physical health status, depression, life satisfaction, and Yangsaeng (health management) of Korean older adults in nursing homes. A quasi-experimental study design using a pretest-posttest control group was employed. Study participants were a total of 80 older adults (intervention: *n* = 40, control: *n* = 40) in a nursing home in Seoul, South Korea. Yangsaeng therapy as an intervention consisted of the Meridian therapy and Qi-gong therapy. Yangsaeng therapy was conducted for 50 min per one time, twice a week, and for 10 weeks. Measures were general characteristics of study participants, Cornell Medical Index, Geriatric Depression Scale, life satisfaction scale, and Yangsaeng scale. Data were collected from April 2018 to March 2019. There were statistically significant differences on physical health status, depression, life satisfaction, and Yangsaeng between the two groups. Yangsaeng therapy was an effective intervention for improving physical health status, life satisfaction, and Yangsaeng, and for decreasing depression of older adults in nursing homes. Health care providers need to pay attention to Yangsaeng therapy as a Korean traditional intervention method for the health management of the older adults residing in nursing homes.

## 1. Introduction

South Korea is a country with the fastest-aging population in the world. In 2018, the older adult population reached 14.3%, thereby approaching an aged society. It is expected to reach 15.7% by 2020 and 24.3% by 2030, thus demonstrating a rapid change toward a super-aged society [1]. In addition to this rapid change in population aging, the number of older adults, who were admitted to nursing homes, tended to increase from 31% in June 2009 to 35.3% in February 2016 among the total number of insured persons since the introduction of long-term care insurance in 2008 [2]. This means that the number of older adults admitted to nursing homes is steadily increasing due to the change in the values of the older adults, change in family structure, increase in women’s social advancement, and change in social awareness [3,4,5].

For the older adults, admission to nursing homes is a serious life event and it can be viewed as a life crisis [5,6]. They may experience a sense of loss due to the notion of an abrupt change from what has been associated with their life so far [3,7]. In particular, it is a more stressful event for the older adults who were admitted to nursing homes regardless of their intentions, and they experience negative emotions, such as worthlessness and helplessness [8,9]. It is common knowledge that it takes an average of six months or more for the elderly to solve problems that occurred in the changed environment after entering the facility and develop close relationships with others [5,6]. Voluntary and involuntary relocation of the older adults may cause ‘Relocation stress syndrome (NANDA-I - North American Nursing Diagnosis-I)’ as they experience many changes in their lifestyle, such as changes in daily life patterns, social networks, and support systems, which had been established for them [5,6].

The older adults residing in nursing homes are suffering from chronic diseases with deterioration of physical function due to aging, and their levels of physical and cognitive functions are significantly weakened as a result of their multiple medications [5,7]. At the same time, the older adults residing in nursing homes are socially and mentally vulnerable because they experience changes in family and social relationships with their family members and friends, and they have limited opportunities to participate in social networks [4,10]. For this reason, the older adults residing in nursing homes may experience depression as a mental disorder, along with physical health-related problems that may result in maladjustment to the facility and lower life satisfaction [4,11].

In the Orient, Yangsaeng (health management) has been proposed as a lifestyle for maintaining and promoting health for longevity [12,13]. Yangsaeng enhances various constitutions, prevents diseases, and increases the lifespan of the older adults; therefore, it can be used as a traditional health care method that can improve their health span [13]. Yangsaeng is keeping a regular lifestyle for the longevity in proper food, exercise, emotion, and sex life to strengthen the body and prevent illness [14]. It includes mentality, foods, living habits, activities and rest, sleep, and sexual life [14]. Those who adhere to the principle of Yangsaeng take good care of their bodies in order to prevent disease [14]. Donguibogam: Principles and Practice of Eastern Medicine explains that humans can enjoy 100 years of life because they have been able to take good care of themselves by eating properly and maintaining a regular life [12,13]. Yangsaeng, which is a traditional health management, can improve the quality of life of the older adults by preventing diseases and increasing their health span [14].

Studies on Yangsaeng show that Korean older adults manage their daily routines in order to prevent aging and improve their health, especially maintaining their physical condition by eating regularly and having proper activities and rest [13]. Therefore, the Korean older adults are expected to have an improved health span through Yangsaeng, which is a traditional health management. In order to promote the healthy life of the rapidly increasing number of older adults residing in facilities and to help them achieve successful aging, research on the development and effectiveness of traditional Korean intervention method to raise the level of Yangsaeng of the older adults residing in facilities is deemed necessary.

However, it was difficult to find the research papers on the Yangsaeng of the older adults residing in nursing homes. In particular, there were no experimental interventional studies applying traditional Korean interventions to the older adults residing in facilities. However, there were some research papers related to traditional Korean medicine for the older adults [12], as well as a small number of experimental studies that showed positive effects on the physical, mental, and psychological indicators [10]. A great deal of attention has been given toward the health-related field for the older adults residing in facilities due to the surge in the older adult population and the increase of the older adults residing in nursing homes. However, the experimental intervention studies are insufficient, and there are no experimental intervention studies to develop and verify the effectiveness of traditional Korean interventions for the health management of the older adults residing in nursing homes. Therefore, the study was conducted to examine the applied effects of Yangsaeng therapy developed through Meridians and Qi-gong therapy to the older adults residing in nursing homes in order to improve their physical health status, reduce their depression, and improve their life satisfaction and Yangsaeng.

The aims of this study were to examine the effects of Yangsaeng (health management) therapy on physical health status, depression, life satisfaction, and Yangsaeng of Korean older adults in nursing home.

## 2. Material and Methods

### 2.1. Study Design and Participants

**Figure 1:**
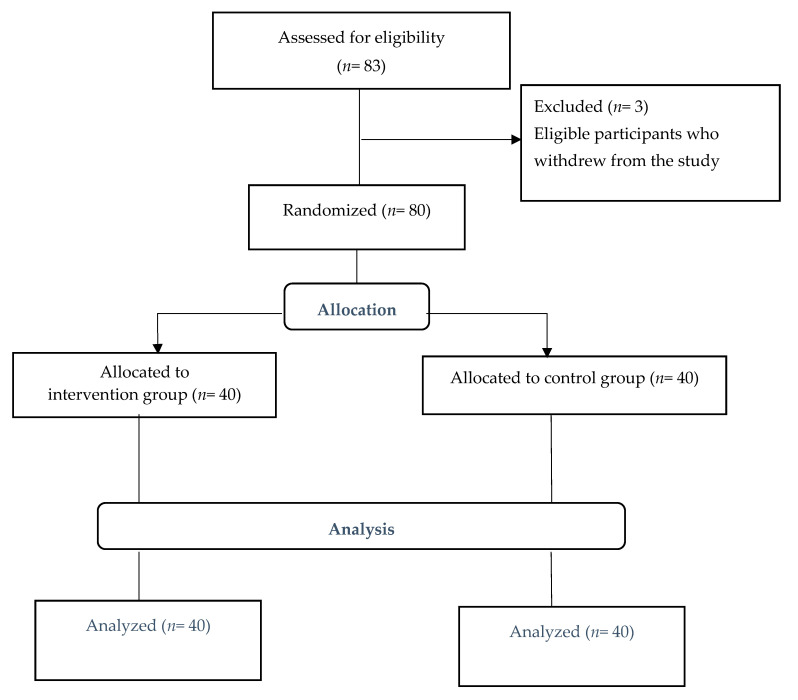
Flowchart of subject progress.

A quasi-experimental study design using a pretest-posttest control group was employed. The study participants were a total of 80 older adults (intervention group: *n*= 40, control group: *n*= 40) in a nursing home in Seoul, South Korea. Study participants participated through convenience sampling in this study. The participants were assigned by randomized assignment method using the coin toss into each group. Eligibility criteria included older adults, 65 years or older, who agreed to take part in it, had a cognitive ability (Mini Mental State Examination, MMSE 23 points or above/30 full marks) to respond, had capability to verbally mutually understand in Korean, and who were able to carry out daily activities (Activities of Daily Living, ADL 24 points or above/30 full marks) in a nursing home. The exclusion criteria included persons who did regularly practice with any exercise, diagnosed by expert. All participants completed the study, and there was no retention or drop out (Figure 1). Sample size adequacy (*n* = 34 in each group) using t test, G power 3.1 analysis software was estimated based on an alpha level of 0.05, medium effect size of 0.5, and power of 0.80 [15]. Therefore, the sample size in this study was appropriate.

### 2.2. Experimental Intervention

Yangsaeng therapy is a one of the interventions in Korean traditional medicine. It is based on Meridian exercise and Pal-dan-gum as Qi-gong therapy. Meridian exercise is an exercise method to hit with palm of the hand on the Meridians flow [13]. Pal-dan-gum as Qi-gong therapy is a therapy including three common basic practices [12,13]. First, relax the whole body and maintain a certain posture. Second, learn and practice the proper way of breathing. Third, induce a state, the so-called freedom from all ideas and thoughts, by concentrating the mind on a specific part of the body. In other words, Pal-dan-gum as Qi-gong therapy is a balance method for correcting the balance of the body with carefulness in conduct, and it is an exercise method for developing the breathing ability with breathing control and maintaining calmness and mental stability [12,16]. Yangsaeng therapy was developed by confirming its reliability and validity by consulting with an experts’ group (a total of 10) consisting of professors of traditional Korean medicine, traditional Korean medical doctors, nursing school professors, nurses in senior nursing homes, and technicians in chiropractic. Yangsaeng therapy includes Meridian exercise and Pal-dan-gum as Qi-gong therapy, and the therapy was applied for 50 min/1 time, 2 times/1 week, and for 10 weeks.

Yangsaeng therapy used for the intervention consisted of greeting for five minutes, the Meridian exercise for 15 min, Qi-gong therapy (Pal-dan-gum) for 20 min, and Meridian exercise (wrap-up) for 10 min (Table 1). Yangsaeng therapy was conducted by one lead researcher and one assistant instructor (Yangsaeng therapy expert) in a nursing home where the participants reside. The instructor who taught Meridian and Qi-gong therapy (Pal-dan-gum) was certified, and the instructor has been educating and serving in the community for many years. For the consistency of the therapy, they first confirmed the consistency of the Yangsaeng therapy movements.

### 2.3. Measures

The study tool package was designed to measure general characteristics of study participants, physical health status, depression, life satisfaction, and Yangsaeng.

Based on a literature review, previous research, and medical or nursing records, a set of general characteristic variables consisted of gender, age, marital status, education, duration of admission, religion, decision maker of admission, drawee of cost, chronic disease, and economic status. This consisted of a total of 10 items.

Cornell Medical Index (CMI) developed by Brodman et al. [17] was standardized for Korean by Nam [18]. This scale was used to measure the level of physical health status of the participants. It was 31 items with four scores on Likert scale. Possible score range was 31–124, and the higher the score was, the higher the level of physical health status. The Korean version of the CMI showed an acceptable to good face and content validity in the previous study [18] and was standardized for Korean. At the time of the development [16], Cronbach’s α = 0.86, and reliabilities in this study were Cronbach’s α = 0.88.

Geriatric Depression Scale (GDS) developed by Sheikh and Yesavage [19] was created into a Korean version by Cho et al. [20]. This scale was used to measure the degree of depression of the participants. It consisted of a total of 15 questions using yes (zero score) or no (one score). The possible score range was 0–15, and the higher the score of the respondent was, the higher the level of depression. Six or more of a total of 15 scores were included into depression state, 0–5 scored into normality in depression. As for the Korean version of the GDS, the scale showed an acceptable to content validity with the discriminant validity being reported as moderate to high [20]. At the time of the creation to the Korean version [20], Cronbach’s α = 0.92, but in this study Cronbach’s α = 0.93.

Life satisfaction scale for Korean developed by Yun [21] was used to measure the level of life satisfaction of the participants. It was 20 items with four scores on a Likert scale. Possible score range was 20–80, and the higher the score was, the higher the level of life satisfaction. About the life satisfaction scale, the scale was validated as Cronbach’s α = 0.93 in the Korean older adults [21]. In the present study, the Cronbach’s alpha coefficients was 0.93.

Yangsaeng scale developed by Kim [22] was used to examine the level of Yangsaeng of the participants. It consisted of a total of 31 questions, using a 5-point Likert scale, subcategorized into five questions on Morality Yangsaeng, four questions on Mind Yangsaeng, five questions on Diet Yangsaeng, four questions on Activities and Rest Yangsaeng, three questions on Exercise Yangsaeng, four questions on Sleep Yangsaeng, three questions on Seasonal Health Yangsaeng, and three questions on Sexual Life Yangsaeng. The most likely score ranged between 31–155, and the higher the score of the respondent, the higher was the level of Yangsaeng. As regarding the Yangsaeng scale, at the time of the development [22], Cronbach’s α = 0.89. In this study, total Cronbach’s α = 0.88, and the Cronbach’s alpha coefficients for eight subscales were as follows: Morality (α = 0.93), Mind (α = 0.87), Diet (α = 0.88), Activities and Rest (α = 0.89), Exercise (α = 0.92), Sleep (α = 0.84), Seasonal Health (α = 0.83), and Sexual Life (α = 0.84).

### 2.4. Procedures

Data were collected from April 2018 to March 2019. After obtaining the IRB (Institutional Research Board) approval from a university for the study, the lead researcher visited the nursing home in order to request research permission from the nursing home where the study participants were residing. After receiving the research permission letter from the institution, the study participants were selected. The purpose of this study, contents and method of experimental intervention, study procedure, questionnaire, measurement variables, etc., were explained to the participants. The study was initiated after the participants voluntarily provided their written informed consent form.

On the first day of the intervention, the research team provided guidance on Yangsaeng therapy with the information on its effectiveness, and physical activity was managed by creating a health handbook in order to induce motivation for intervention participation. Yangsaeng therapy was applied by the lead researcher and an assistant to the intervention group. As a reward, different success souvenirs were provided according to the achievement rate, as compared to the intervention goal. For the control group, there was no treatment provided for the purpose of intervention, and the participants were guided to lead the same daily life as before. Pretest and posttest were conducted by the other research assistant in the nursing home where the Yangsaeng therapy was provided. The research assistant read the self-report questionnaire to the participants on a one-to-one basis and requested them to fill out the questionnaire. Participants with difficulty in understanding filled out the questionnaire with the help of the research assistant. Only 15 participants had difficulties in filling out the questionnaires. Each of the participants took approximately 25–30 min to complete the questionnaire. After the study finished, the same Yangsaeng therapy was provided for the participants in the control group as an ethical consideration.

### 2.5. Statistical Analysis

The collected data were analyzed using the SPSS version 21.0 statistical software program. General characteristics of the study participants and homogeneity test were analyzed using descriptive statistics with frequency, percentage, mean, and standard deviation. For homogeneity test, *t*-test, χ^2^ test, and Fisher exact test were used to examine the effects of Yangsaeng therapy and were analyzed using an independent *t*-test. A *p*-value of less than 0.05 was considered statistically significant.

### 2.6. Ethical Considerations

In the ethical consideration, approval for this study was obtained from IRB committee in a university (GU-201806-HRa-01-02). The purpose and contents of the study, anonymity of the participants, and information on confidentiality were explained, and a written informed consent form was obtained from the participants who voluntarily wanted to participate in the study. The participants were told that their study participation was voluntary and that they were allowed to withdraw their participation at any time during the study. In addition, there was no harm caused by their withdrawal in the middle of the study.

## 3. Results

### 3.1. General Characteristics of the Study Participants and Homogeneity

The majority of the participants were female (intervention: 75.0%, control: 72.5%). The average age was 83.68 years (intervention: 83.18 years, control: 84.18 years). As for the marital status, most of the participants were widowed (intervention: 77.5%, control: 77.5%). Most were elementary school graduates or below (intervention: 67.5%, control: 60.0%). Regarding the duration of admission, most of the participants had been at the nursing home for 3 to 5 years (intervention: 47.5%, control: 42.5%). As for the decision maker of admission, participants reported that child(ren) was (were) 27.5% in intervention group, and 25.0% in control group. In terms of the drawee of cost, the child(ren) was (were) the most (intervention: 70.0%, control: 60.0%). Also, most of the participants had chronic disease (intervention: 72.5%, control: 70.0%). As for the general characteristics of the intervention and control groups, there were no group differences at baseline at a statistical significance level of *p* < 0.05 (Table 2).

There were no differences between the intervention group and control group on the physical health status (t = 0.175, *p* = 0.862), depression (t = 1.282, *p* = 0.204), life satisfaction (t = 0.000, *p =* 1.000), and Yangsaeng (t = −0.869, *p* = 0.388) of study participants. Therefore, as for the study variables before the intervention, there were no group differences at baseline at a statistical significance level of *p* < 0.05 (Table 3).

### 3.2. Effects of Yangsaeng Therapy

The physical health status (t = 10.046, *p* < 0.001), depression (t = −13.191, *p* < 0.001), life satisfaction (t = 19.256, *p* < 0.001), and Yangsaeng (t = 8.644, *p* < 0.001) of study participants were more improved in the intervention group than in the control group. In addition, Morality, Mind, Diet, Activities and Rest, Exercise, Sleep, Seasonal Health, and Sexual Life as subcategories all in Yangsaeng were improved (Table 4).

## 4. Discussion

The average age of the subjects was 83.68 years and the majority were 75 to 84 years old. For most, the marital status was widowed. The most common duration of stay was three to five years, which was similar to the study results of Kim [23] and Lim et al. [24]. Most of the people who decided the admission of the older adults to the facility were their children and spouses, and most of the people who paid for the stay of the older adults in the facility were their children and the elderly themselves. This was similar to the study results of Shin et al. [11] and Kang et al. [9]. It was found that the decision maker and payer for the Korean older adults residing in nursing homes were mostly the children of the elderly. It seems that the Korean society still has a sense of traditional extended family culture and a sense of respect for the parents and responsibility as children in their old age, despite a lot of nuclear families.

The physical health status of the participants was slightly higher than the median of 77.5 in both the experimental and control groups, which was very similar to the results of the previous study [10]. It seems that the older adult participants could perform their daily living activities in the nursing homes [3,25]. The mean depression score was higher than 8 points in both groups, thereby indicating that the older adult participants were in a depressed state. This result was similar to those of the previous studies [11,25], thereby suggesting that the older adults participants were depressed due to their relocation to the nursing homes, stress from unfamiliar surroundings, and reduced intimacy with their family members [9,24]. Life satisfaction was slightly higher than the median of 50 in both groups, thereby showing a different result when compared with the previous study [26]. As life satisfaction was found to be slightly higher in the depressed state in this study, it was necessary to confirm it through further research. The level of Yangsaeng of the older adult participants was lower than the median of 93 in both groups, which was similar to the results of the previous study [14] on health management-related variables. This suggests that the health management level of the older adults residing in nursing homes is relatively low.

In this study, the Yangsaeng therapy improved physical health status, life satisfaction, and level of Yangsaeng, as well as reduced the depression for the older adults residing in nursing homes, thereby showing similar results to that of the previous studies [12,16]. It showed that Yangsaeng therapy had positive effects on the physical health status, depression, life satisfaction, and level of Yangsaeng of the older adults residing in nursing homes. Also, all subcategories (Morality, Mind, Diet, Activities and Rest, Exercise, Sleep, Seasonal Health, and Sexual Life) in Yangsaeng were improved.

Yangsaeng therapy is based on Meridian therapy that stimulates the body’s acupuncture points and Meridians, while at the same time unites the energy and blood through slow breathing [13,16]. It releases extravagated blood and malicious energy through exhalation and skin, relaxes even the small muscles deep inside the body, and activates the function of the internal organs to keep the head cool and the organs warm [10]. It also corrects the cervical spine, spine, and pelvis, and prevents stagnation of the energy in the joints [16]. These positive effects brought about by the physiological principles of Meridian therapy and Qi-gong therapy were found to be effective in the older adults residing in nursing homes [12,13].

Based on the findings of this study, the Yangsaeng therapy consisting of Meridian exercise and Pal-dan-gum as Qi-gong therapy can be utilized as a health management method for reducing the degree of depression and improving the physical health status, life satisfaction, and level of Yangsaeng of the older adults residing in nursing homes. Furthermore, it is necessary to recruit and manage specialists who can apply Yangsaeng therapy to the older adult in nursing homes, and policies should be established so that it can be carried out regularly. Repeated experimental interventional studies are deemed necessary in order to determine whether Yangsaeng therapy can be applied and used in the community, such as senior welfare centers, senior schools, and religious organizations.

The findings from this study are difficult to generalize because study participants were conveniently extracted from and older adults’ nursing home located in some area. Additionally, during the period of applying the Yangsaeng therapy as an experimental intervention method, the individual exercise that individual participants performed was not completely controlled. This may be a limitation of the study.

## 5. Conclusions

In this study, Yangsaeng therapy improved the physical health status of the older adults residing in nursing home, decreased their degree of depression, and enhanced their life satisfaction and level of Yangsaeng. Health professionals will be able to apply it practically in Yangsaeng therapy as an intervention method for the health management of the older adults residing in nursing homes. In the current situation, where there is a lack of experimental interventional studies to develop and verify the effectiveness of Korean traditional interventions for the health management of the older adults residing in nursing homes, this study has as its significance that it applied a Korean traditional intervention called Yangsaeng therapy, and verified and analyzed its effectiveness. The preliminary findings will be strengthened by replicating this study with a larger sample. In addition, more research into this method is needed.

## Figures and Tables

**Table 1:** Contents of Yangsaeng therapy.

Order (Time)	Contents
Greeting (5 min)	Preparation for gymnastic formation, checking the surroundings, installing safety equipment
Meridian exercise (15 min)	- Through Baeghwe point, Yongcheon point, and Injung point, do abdominal breathing a total of 30 times. - With your fingertips, lightly tap the meridian points on the head from the Baeghwe point. - Move down to the face and tap lightly the meridian points around the forehead, cheeks and eyes. - Press hard on the eyebrows. Then press firmly on the bone protrusion under the eye. - With the index fingers of both hands, Press firmly on the inner corners of the eyes (Cheongmyung Point). - Strongly press the Taeyang Point, hardly push the Amun Point, and also firmly press the Pungji Point. - Massage from Amun Point and Pungji point to the shoulder areas with your fingers pinching. - Hit the chest several times vigorously, and stretch your left arm and hit down the inside of your left arm with your palm. The palms are facing each other and the palm coming back to the chest while vigorously hitting the outside of your left arm. Hit the chest several times and do the same motions on the right arm as on the left. - After hitting the center of both sides of the chest, going down to the ankles vigorously while hitting. And comes up from the inner leg to the chest again. Descending from the chest to the side of the leg and rising from the inner leg to the chest, from the back waist to the back ankle, and from the inner leg to the chest. - Gently stroke the chest and abdomen. - Keep the legs straight and make the toes straight. Then hit the right and left toes 20 times each other. - Clap 20 times. - Sit up and do abdominal breathing 30 times as in the previous exercise.
8 Brocades: Qi-gong therapy (Pal-dan-gum) (20 min)	- First Brocade: Two hands hold up the heavens - Second Brocade: Drawing the bow to shoot the eagle - Third Brocade: Separate heaven and earth - Forth Brocade: Wise owl gazes backwards or look back - Fifth Brocade: Stay the head and shake the tail - Sixth Brocade: Two hands hold the feet to strengthen the kidneys and waist - Seventh Brocade: Clench the fists and glare fiercely (or angrily) - Eighth Brocade: Lifting up the heels
Meridian exercise (wrap up) (10 min)	- Keep the legs straight and make the toes straight. Then hit the right and left toes 20 times each other. - Clap 20 times. - Sit up and do abdominal breathing 30 times as in the previous exercise.

**Table 2:** General characteristics of study participants and homogeneity.

Characteristics	Intervention Group (*N* = 40) *n* (%)	Control Group (*N* = 40) *n* (%)	t/χ^2^	*p*
Gender				
Male Female	10 (25.0) 30 (75.0)	11 (27.5) 29 (72.5)	0.065	0.799
Age (year)				
65~74 75~84 85~94 95≤ Mean ± SD	2 (5.0) 23 (57.5) 12 (30.0) 3 (7.5) 83.18 ± 5.68	1 (2.5) 20 (50.0) 16 (40.0) 3 (7.5) 84.18 ± 5.27	1.114 † −0.816	0.774 0.417
Marital status				
Married Bereavement	9 (22.5) 31 (77.5)	9 (22.5) 31 (77.5)	0.000	1.000
Education				
None Elementary school Middle school High school	13 (32.5) 14 (35.0) 6 (15.0) 7 (17.5)	12 (30.0) 12 (30.0) 9 (22.5) 7 (17.5)	0.794	0.851
Duration of admission (year)				
1> 1~<3 3~5	13 (32.5) 8 (20.0) 19 (47.5)	11 (27.5) 12 (30.0) 17 (42.5)	1.078	0.583
Religion				
None Protestant Catholic Buddhist	4 (10.0) 14 (35.0) 5 (12.5) 17 (42.5)	3 (7.5) 14 (35.0) 9 (22.5) 14 (35.0)	1.576 †	0.665
Decision maker of admission				
Spouse Child(ren) Brother/Sister Relative Neighbor Pastor/nun/monk	10 (25.0) 11 (27.5) 9 (22.5) 4 (10.0) 2 (5.0) 4 (10.0)	7 (17.5) 10 (25.0) 10 (25.0) 6 (15.0) 3 (7.5) 4 (10.0)	1.149 †	0.886
Drawee of cost				
Spouse Child(ren) Self	2 (5.0) 28 (70.0) 10 (25.0)	2 (5.0) 24 (60.0) 14 (35.5)	0.974 †	0.614
Chronic disease				
No Yes	11 (27.5) 29 (72.5)	12 (30.0) 28 (70.0)	0.061	0.805
Economic status				
High Moderate Low	1 (2.5) 34 (85.0) 5 (12.5)	1 (2.5) 32 (80.0) 7 (17.5)_	0.394 †	0.821

^†^ Fisher exact test.

**Table 3:** Homogeneity of study variables at the pre-intervention.

Scales	Intervention Group (*N* = 40) Mean ± SD	Control Group (*N* = 40) Mean ± SD	t	*p*
Cornell Medical Index (CMI)	78.85 ± 9.78	78.48 ± 9.42	0.175	0.862
Geriatric Depression Scale (GDS)	8.75 ± 1.13	8.45 ± 0.95	1.282	0.204
Life satisfaction scale	56.17 ± 6.44	56.17 ± 5.99	0.000	1.000
Yangsaeng scale	84.25 ± 6.60	85.50 ± 6.25	−0.869	0.388

**Table 4:** Effects of Yangsaeng therapy.

Scales	Intervention group (N = 40)			Control group (*N* = 40)			t *p*
	Pre Mean ± SD	Post Mean ± SD	Difference Mean ± SD	Pre Mean ± SD	Post Mean ± SD	Difference Mean ± SD	
Cornell Medical Index (CMI)	78.85 ± 9.78	104.15 ± 13.53	25.30 ± 17.29	78.48 ± 9.42	73.85 ± 8.97	−4.63 ± 2.55	10.046 <0.001 *
Geriatric Depression Scale (GDS)	8.75 ± 1.13	4.80 ± 1.67	−3.95 ± 2.05	8.45 ± 0.95	10.98 ± 2.09	2.53 ± 1.33	−13.191 <0.001 *
Life satisfaction scale	56.17 ± 6.44	82.50 ± 8.47	26.33 ± 9.25	56.17 ± 5.99	51.68 ± 5.95	−4.49 ± 2.16	19.256 <0.001 *
Yangsaeng scale	84.25 ± 6.60	104.75 ± 17.63	20.50 ± 19.27	85.50 ± 6.25	77.75 ± 6.86	−7.75 ± 5.46	8.644 <0.001 *
Morality	14.12 ± 6.23	15.14 ± 8.72	1.02 ± 2.23	14.36 ± 6.12	13.62 ± 6.23	−0.74 ± 0.35	7.254 0.021 *
Mind	10.24 ± 5.76	13.26 ± 8.12	3.02 ± 2.76	10.32 ± 6.01	8.16 ± 6.17	−2.16 ± 0.16	12.522 <0.001 *
Diet	13.63 ± 6.09	15.64 ± 7.88	2.01 ± 1.65	13.72 ± 6.12	13.43 ± 6.93	−0.29 ± 0.37	10.126 0.001 *
Activities and rest	10.47 ± 5.17	13.93 ± 7.65	3.46 ± 2.62	10.64 ± 6.23	9.17 ± 6.34	−1.47 ± 0.45	9.842 <0.001 *
Exercise	8.46 ± 6.42	12.28 ± 8.16	3.82 ± 2.46	9.13 ± 6.32	7.86 ± 6.76	−1.27 ± 0.42	6.485 <0.001 *
Sleep	10.79 ± 6.15	13.32 ± 8.23	2.53 ± 2.12	10.54 ± 6.02	9.18 ± 6.83	−1.36 ± 0.14	11.423 <0.001 *
Seasonal health	8.42 ± 6.18	10.94 ± 7.76	2.52 ± 1.85	8.37 ± 6.32	8.12 ± 6.94	−0.25 ± 0.25	7.158 0.013 *
Sexual life	8.12 ± 6.42	10.24 ± 7.24	2.12 ± 1.23	8.42 ± 6.12	8.21 ± 6.03	−0.21 ± 0.12	19.256 0.043 *

* *p* < 0.05.

## References

[1] Korea National Statistical Office (2018). Statistics of Population in the Future.

[2] National Health Insurance Korea (2016). Intergraded Nurse Care Service 2016.

[3] Kim M.S. (2018). The influence of self-efficacy and activities of daily living on depression among elderly nursing home residents. J. Korean Acad. Soc. Nurs. Educ..

[4] Koppitz A.L., Dreizler J., Altherr J., Bosshard G., Naef R., Imhof L. (2017). Relocation experiences with unplanned admission to a nursing home: A qualitative study. Int. Psychogeriatr..

[5] Lee H.K., Kim S.Y., Sok S.R. (2016). Effects of multivitamin supplements on cognitive function, serum homocystein level, and depression of Korean older adults with mild cognitive impairment in care facilities. J. Nurs. Scholarsh..

[6] Altintas E., De Benedetto G., Gallouj K. (2017). Adaptation to nursing home: The role of leisure activities in light of motivation and relatedness. Arch. Gerontol. Geriatr..

[7] Meeks S., Van Haitsma K., Mast B.T., Arnold S., Streim J.E., Sephton S., Smith P.J., Kleban M., Rovine M. (2016). Psychological and social resources relate to biomarkers of allostasis in newly admitted nursing home residents. Aging Ment. Health.

[8] Cho E., Kim H., Kim J., Lee K., Meghani S.H., Chang S.J. (2017). Older adult residents’ perceptions of daily lives in nursing homes. J. Nurs. Scholarsh..

[9] Kang E.Y., Chong S.M., Chong B.H. (2018). The relationship between the quality of sleep and the cognitive function, depression, and activities of daily living in the institutionalized elderly. Neurotherapy.

[10] Chang E.K., Park H.K. (2018). Effects of Auricular Acupressure Therapy on Musculoskeletal Pain, Depression and Sleep of the Elderly in Long-term Care Facilities. J. Korean Acad. Commun. Health Nurs..

[11] Shin M.W., Ahn K.S., Cho Y.C. (2017). Factors related to quality of life in the elderly people in long-term care centers. J. Korea Acad. Ind. Cooper. Soc..

[12] Park K.S., Jeong H.Y., Kim Y.H. (2016). The effects of Qi-gong exercise on the health of the elderly-with respect to the physical health status, the fear of falling, balance efficacy, and Hwa-Byung. J. Orient. Neuropsychiatr..

[13] Shin H.S., Sok S.R., Yoo J.H., Im Y.J., Jang M.H., Jang A.K., Jang A.K., Jeong Y.H., Kang Y.M., Kim Y.J. (2018). Understanding and Practice in Oriental Nursing: Kyung Hee University, College of Nursing Science.

[14] Gu M.K., Sok S.R. (2018). Predictors of Yangsaeng (health management) among Korean middle-aged adults. Holist. Nurs. Pract..

[15] Faul F., Erdfelder E., Lang A.G., Buchner A. (2007). G power 3: A flexible statistical power analysis program for social, behavioral, and biochemical sciences. Behav. Res. Method.

[16] Ahn Y.J., Jo S.H., Lee S.H., Lim J.H. (2016). The review study on Yoga, Qigong, and Tai Chi interventions for anxiety: Based on Korean journal articles from 2009 to 2015. J. Orient. Neuropsychiatr..

[17] Brodman K., Erdmann A.J., Lorge I., Wolff H.G., Broadbent T.H. (1951). The Cornell Medical Index-Health Questionnaire. II. As a diagnostic instrument. J. Am. Med. Assoc..

[18] Nam H.C. (1965). Study on the Cornell Medical Index.

[19] Sheikh J.I., Yesavage J.A. (1986). Geriatric depression scale (GDS), recent evidence and development of shorter version. Clin. Gerontol..

[20] Cho M.J., Bae J.N., Suh G.H., Hahm B.J., Kim J.K., Lee D.W., Kang M.H. (1999). Validation of geriatric depression scale (GDS), Korean version in the assessment of DSM-III-R major depression. J. Korean Neuropsychiatr. Assoc..

[21] Yun J. (1982). A Study of Tool Development for Living Satisfaction of Elderly.

[22] Kim A.J. (2004). Development of a tool in measuring Yangsaeng. J. Korean Acad. Nurs..

[23] Kim J.I. (2015). Prediction of quality of life among the elderly at care facilities for the elderly according to health states, physical and cognitive functions, and social supports-focused on D metropolitan city. J. Korea Acad. Ind. Cooper. Soc..

[24] Lim Y.A., Shin T.S., Cho Y.C. (2018). The association of physical and mental function with quality of life among the elderly at care facilities. J. Korea Acad. Ind. Cooper. Soc..

[25] Kim H.O., Kim S.H., Oh U.L., Han U.L., Jeong A.R., Ju Y.E. (2019). Impacts of Activities of Daily Living and Depression on the Meaning of Life of Alder Adults in Nursing Home.

[26] An S.H. (2019). The effects of exercise program on cognitive function, depression, and life satisfaction in elderly. J. Digit. Converg..

